# The Effect of the Color Filter Array Layout Choice on State-of-the-Art Demosaicing

**DOI:** 10.3390/s19143215

**Published:** 2019-07-21

**Authors:** Ana Stojkovic, Ivana Shopovska, Hiep Luong, Jan Aelterman, Ljubomir Jovanov, Wilfried Philips

**Affiliations:** TELIN-IPI, Ghent University—imec, 9000 Ghent, Belgium

**Keywords:** demosaicing, debayering, color filter array, image interpolation, image reconstruction

## Abstract

Interpolation from a Color Filter Array (CFA) is the most common method for obtaining full color image data. Its success relies on the smart combination of a CFA and a demosaicing algorithm. Demosaicing on the one hand has been extensively studied. Algorithmic development in the past 20 years ranges from simple linear interpolation to modern neural-network-based (NN) approaches that encode the prior knowledge of millions of training images to fill in missing data in an inconspicious way. CFA design, on the other hand, is less well studied, although still recognized to strongly impact demosaicing performance. This is because demosaicing algorithms are typically limited to one particular CFA pattern, impeding straightforward CFA comparison. This is starting to change with newer classes of demosaicing that may be considered generic or CFA-agnostic. In this study, by comparing performance of two state-of-the-art generic algorithms, we evaluate the potential of modern CFA-demosaicing. We test the hypothesis that, with the increasing power of NN-based demosaicing, the influence of optimal CFA design on system performance decreases. This hypothesis is supported with the experimental results. Such a finding would herald the possibility of relaxing CFA requirements, providing more freedom in the CFA design choice and producing high-quality cameras.

## 1. Introduction

Since Bayer’s original patent [1], (Bayer) Color Filter Array (CFA) demosaicing has established itself as the de facto standard method of acquiring multi-dimensional color images. General demosaicing in this sense would be defined as the reconstruction of a (multi-dimensional) color signal from an inherently single-dimensional array of (e.g., Charge-Coupled Device (CCD) or Complementary Metal-Oxide Semiconductor (CMOS)) sensors. The mosaiced image is obtained by using a planar sensor that is covered by an interleaved pattern of different color filters, resulting in sensor output that is an interleaved pattern of signal components that represent different parts of the color spectrum. A demosaicing algorithm reconstructs this into a (three-dimensional) full color signal. An optimal demosaicing system design would be constituted of the creation of an optimal interleaving pattern (the color filter array or CFA) and an optimal demosaicing algorithm that achieves the highest color reconstruction quality.

### 1.1. CFA Pattern Design

A good CFA pattern design satisfies the criteria presented in [2]: cost-effective image reconstruction, robustness to color aliasing, robustness to image sensor imperfections and robustness to optical or electrical influence between the neighboring pixels. The Bayer CFA exploits the fact that the human eye is more sensitive to wavelengths corresponding to the green colors, rather than to the blue and the red colors, i.e., the Bayer CFA consists of a repeating 2 × 2 pattern of one red, one blue and two green color filters. Given the analysis for the spectra of different CFA patterns, performed by Hirakawa et al. and presented in [3], it can be concluded that the Bayer CFA pattern is susceptible to introducing aliasing artifacts in both vertical and horizontal high spatial frequencies. Therefore, other solutions for CFA design that will overcome the problems with aliasing artifacts introduction for horizontal and/or vertical edges, were proposed. The idea for these designs is either based on the assumption that the physical world is rather horizontally than vertically oriented, or on the opposite assumption. Examples for more oriented patterns are: the Yamanaka CFA pattern [4], the Lukac CFA pattern [2], the Vertical CFA pattern, the Modified Bayer CFA pattern, the Diagonal CFA pattern, etc. With a purpose to increase the quality of the low-light photography, Sony introduced the Quad Bayer CFA sensor. In order to reduce the sensitivity to noise, many camera systems use multi-frame photography, which leads to ghosting artifacts introduction that affects the quality of the captured video. To deal with the problem of ghosting artifacts introduction, Sony introduced the IMX586 smartphone sensor with the Quad Bayer design and 48 MP resolution [5], with which Sony succeeds to obtain high performance and quality gain (as explained in [6]) when used in the high dynamic range (HDR) mode in low-light conditions. According to Sony, in normal light conditions, the camera achieves a similar quality of the captured images, when these are compared with the images captured with a sensor of 12 MP sensor with the Bayer design. Furthermore, to deal with the problem of low sensitivity in low-light conditions, many panchromatic CFA designs were introduced, e.g., Sony 4-Color [7], Kodak Ver.1-3 [8], etc. There also exist works on the multispectral filter array design [9,10] that find application in different fields of multispectral imaging.

In our analysis, we will only test the influence of Bayer-like CFA patterns (with the same sampling ratio as the Bayer CFA), considering them to be sufficient to show that the difference in quality performance between different CFA designs decreases with the increasing power of demosaicing algorithms.

### 1.2. Demosaicing

Since demosaicing has been an extensively studied research area, it abounds with algorithms that may be classified into different groups. The earliest works are based on using simple interpolation techniques (bilinear, bicubic, spline interpolation, etc.). These reconstruction techniques are prone to artifacts introduction (aliasing) in the regions with high spatial frequencies, i.e., regions with edges and details. Consequently, the research in this field progressed further towards designing algorithms based on more sophisticated reconstruction techniques. For that purpose, numerous survey studies have been proposed [11,12,13]. In [14], the demosaicing algorithms were roughly classified into five categories, depending on the used techniques and on the reconstruction domain (spatial, frequency, wavelet, etc.). According to this classification, *classical* demosaicing includes: frequency-domain algorithms (good representatives are [15,16]), algorithms based on directional interpolations (among which residual interpolation (RI) algorithms, specifically [14] show superior performance), wavelet-based algorithms (among which good representatives are the algorithms presented in [17,18,19]), and reconstruction-based (with the algorithms presented in [20,21] as good representatives of this group). Another group of algorithms are the *learning-based* algorithms: dictionary-learning-based [22], regression-based [23], reconstruction-based with machine learning procedures for multispectral demosaicing (with [24] being a good representative algorithm) and neural-network-based algorithms, which we will refer to as *modern learning-based* demosaicing algorithms (with [25] as a representative).

**Figure 1:**
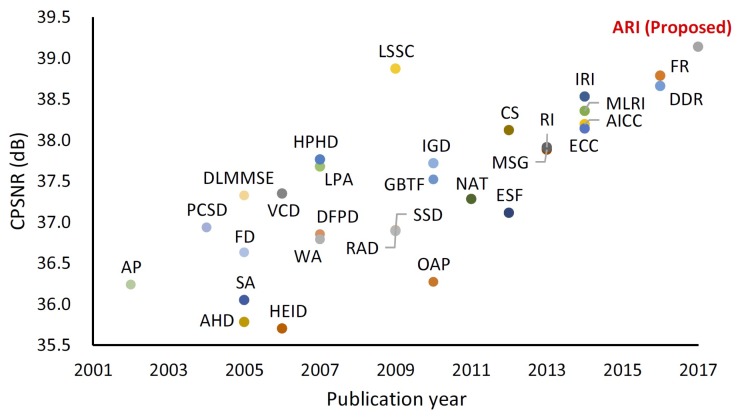
Performance of the demosaicing algorithms over the years (from the analysis presented in [14]). Image source: [14].

A good overview of the performance of the *classical* demosaicing algorithms (that are not deep-learning-based) is given in the graph from [14], shown in Figure 1. As it can be seen, ARI [14] outperforms all previous algorithms. Its good performance is due to the fact that it is an iterative approach based on the assumption of color consistency along the edges and smooth areas. Additionally, the algorithm considers the color differences in creating the final demosaiced image. However, this method, despite its good performance in the way it was designed, is not generic and is applicable only to Bayer pattern CFA and the multispectral filter array (MSFA) that was presented earlier in [10]. *Classical* demosaicing techniques have an advantage of being applicable to any type of image without requiring training data, while, on the other hand, the *modern learning-based* algorithms show superior performance and can be modified to be applied on different CFA patterns, which makes them generic.

### 1.3. CFA-Demosaicing Co-Design

The co-design of CFA and demosaicing has received little attention. A reason for this is that many *classical* demosaicing techniques are intricate interpolation schemes that are finely tuned to a particular CFA pattern, typically the ubiquitous Bayer pattern. The intricacy of this design precludes the possibility of applying a demosaicing algorithm to a different CFA pattern and therefore inhibits the application of well-performing demosaicing techniques to CFA patterns that they were not designed for. There exist approaches in which a particular CFA design is introduced and a matching demosaicing algorithm is proposed (e.g., Pseudo-randomized CFA pattern [26] design and a demosaicing algorithm presented in [20]). Similarly, in [2], a Bayer-like CFA design is proposed and a generic algorithm is devised [27]. Demosaicing algorithms that are generic, in a sense that may be applied successfully to any CFA, do exist, but are much less common and are typically outperformed by CFA-specific demosaicing. Examples of such algorithms are [20,21], which belong to an earlier generation of demosaicing algorithms. These algorithms were outperformed by ARI (for the Bayer CFA pattern) and by another state-of-the-art algorithm, known as ACUDe [28] that belongs to the group of classical demosaicing methods and is also generic.

Another generic algorithm that belongs to the group of newer generation of *classical demosaicing* algorithms is the algorithm presented in [29], where the authors for reconstruction use the linear minimum mean square error (LMMSE) model. The LMMSE approach was tested on different periodic RGB CFA patterns and the experimental analysis has shown that, for some random CFA patterns, it achieves better performance than for the Bayer CFA pattern. Furthermore, with this analysis, it was shown that, for all analyzed CFA patterns, the reconstruction quality increases as a bigger neighborhood around the pixel to be estimated is taken into consideration.

More advanced generic algorithms are the learning based algorithms, among which neural network based algorithms are superior. Such an algorithm is presented in [30]. Here, authors propose a demosaicing algorithm based on a simple neural-network architecture. In this algorithm, the authors rely on the previously proposed concept of using a superpixel (neighboring area) for estimating the unknown pixel values. This technique was tested on different CFAs and the MSFA presented in [10].

Convolutional neural networks (CNNs) have shown great success in many image processing and computer vision tasks. One of the main strengths of CNNs is that they allow learning features specific for a given domain, compared to earlier approaches where the features were predefined based on domain knowledge. For example, for the problem of demosaicing, the same CNN can be applied for different CFAs by retraining it with different input data, without any structure modifications [25,31,32,33,34,35,36]. Moreover, some CNN-based algorithms learn an optimal color filter layout jointly with a model for demosaicing of images obtained with the learned pattern [37,38].

### 1.4. Structure

This paper studies the effect of the CFA design choice on the overall CFA-demosaicing reconstruction quality. Specifically, we test the hypothesis that the impact of the CFA layout choice on the quality performance decreases with the emergence of more sophisticated, *modern learning-based* demosaicing algorithms.

It is structured as follows: in Section 2, we provide a description of demosaicing that is state-of-the-art with respect to quality performance and that lends itself well to adaptation and improvement towards generic CFA. In Section 3, we describe adaptations we made to these methods in an effort to allow evaluation of different CFAs and to achieve better results in terms of reconstruction quality. In Section 4, by starting with the motivation for the performed analysis, we proceed with explanation about the performed experiments, about the used image data-sets and the used CFA patterns. In Section 5, we present the qualitative and the quantitative results from the performed analysis, while in Section 6 we give a summary of the obtained conclusions.

## 2. State-of-the-Art Demosaicing

In order to demonstrate a trend of state-of-the-art demosaicing becoming less sensitive to the CFA pattern (i.e., to show that the performance of *modern learning-based* demosaicing is less affected by the CFA design), we selected two algorithms, as representatives of two different groups of demosaicing algorithms: in the first group, we consider *classical* demosaicing techniques that are not based on machine learning and in the second group we consider *modern learning-based* techniques. In this section, we describe the two representatives of each group separately. Specifically, the first algorithm, known as ACUDe, belongs to the group of directional interpolation demosaicing algorithms. For reconstructing the full color image, it exploits the color consistency in real images, by using the interchrominance dependency. The second algorithm is a neural network based algorithm called CDMNet [25], chosen as a representative method with a publicly available code.

### 2.1. Universal Demosaicing of CFA (ACUDe)

The method proposed by Zhang et al. [28], known as ACUDe, is devised to be applied on different designs of CFA. Considered to be a state-of-the-art generic algorithm among the algorithms that are based on directional interpolations, it has similar performance to the state-of-the-art ARI algorithm proposed by Monno et al. [14], which outperforms all demosaicing algorithms that were previously proposed. Furthermore, as ARI was implemented, it is only applicable to the Bayer CFA pattern and the five-band multispectral filter array described in [10,14]. Therefore, we will consider only ACUDe for our analysis. Although the main idea and theory of ACUDe can be applied on multispectral filter arrays, the algorithm was tested and implemented for the three primary color system. Considered to be an adaptive generic method, it exploits the inter-chrominance dependence and the measured CFA response, to estimate the chrominance components. It is implemented in three steps: estimation of the color/demosaicing transform matrix (only dependent from the CFA pattern) and the chrominance direction; distance dependent per-pixel weight generation based on inter-pixel chrominance capture and edge sensitivity; chrominance components estimation by weighting over the CFA image and demosaicing transformation to the three primary colors. The method was evaluated on the KODAK data-set [39] (because it abounds with images with both high and low spatial frequency content) and the IMAX data-set [40] (considered to be more challenging for demosaicing methods because of its high color diversity and also because many images from the data-set contain high spatial frequencies in the chrominance components). In [28], the authors compared their algorithm with two other generic demosaicing algorithms for RGB CFA patterns (Condat’s generic method [20] and Menon and Galvagno’s regularization approach to demosaicing (RAD) [21]). The obtained results (in terms of measured color peak signal-to-noise ratio (CPSNR) and CIE LAB error) indeed show that ACUDe outperforms the two mentioned algorithms for six different CFA patterns.

### 2.2. Demosaicing Using a CNN (CDMNet)

The baseline neural-network-based algorithm in this work is CDMNet [25]. In our analysis, we use this algorithm because of its high-quality performance and reconstruction power among the algorithms that belong to the group of *modern learning-based* demosaicing techniques and because our experiments show that it outperforms the state-of-the-art algorithms that belong to the group of *classical* demosaicing. Furthermore, this algorithm with the publicly available code and the retrainable neural-network is suitable for performing modifications towards making it generic (adaptive to any repetitive CFA pattern). In its original design, CDMNet is a three-stage neural network that is among the state-of-the-art, according to evaluations on different datasets. CDMNet relies on the inter-channel correlation for interpolation of the missing values. The problem of RGB demosaicing is split into three stages: (1) reconstruction of the green channel, (2) separate reconstructions of the red and blue channels jointly with the high-quality green channel and (3) joint fine-tuning of all three channels.

## 3. Modifying State-of-the-Art Demosaicing Algorithms

Here, we present the modifications we made on ACUDe and CDMNet. Since ACUDe is designed to be generic, with our modifications, we achieved slight improvement in the reconstruction quality. The adaptations made on CDMNet apply to the generalisation of this algorithm towards repetitive RGB CFA patterns.

### 3.1. Modifying ACUDe

**Figure 2:**
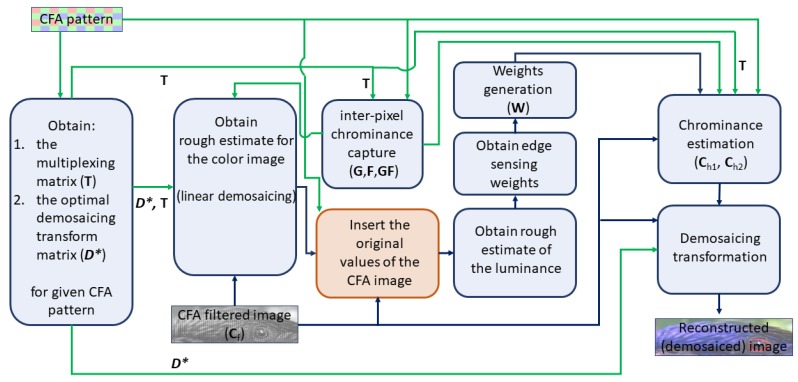
Flowchart of ACUDe and our modifications. Modifications are represented with the procedure in the orange block. Green arrows represent the data-flow between procedures (given with blocks) where only the color filter array (CFA)pattern is used. Blue arrows represent the data-flow between procedures (given with blocks) where also a CFA input image is used.

The flowchart of the modified ACUDe is presented in Figure 2. The input data that is considered is: the CFA pattern and the CFA filtered image (CFA input). The green arrows represent the CFA pattern dependent data-flow, and the blue arrows represent the CFA filtered image dependent data-flow. For more details about the algorithm and each procedure, we refer to the explanation given in [28]. The modifications that were made consist of inserting the original values of the CFA filtered image to the corresponding locations in the output image. This procedure is applied after obtaining the rough estimate of the demosaiced image (non-adaptive demosaicing). For the main source-code used in our implementation and the obtained results for Bayer pattern, of the originally designed (as explained in [28]), and unmodified ACUDe, we direct the reader to the following web-site [41].

### 3.2. Making CDMNet Generic

**Figure 3:**
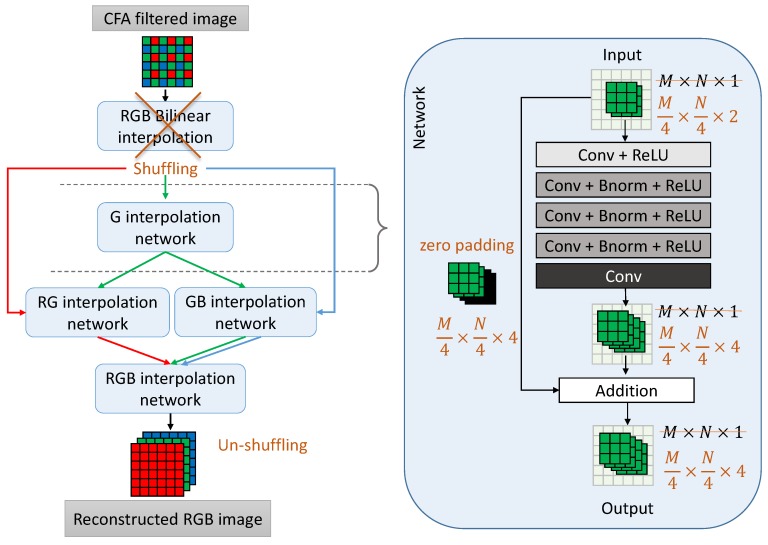
Flowchart of CDMNet and our modifications. The orange color represents locations where we modified the original algorithm to make it agnostic of any CFA pattern: no bilinear demosaicing, pixel shuffling and working in a lower spatial resolution with fixed downscaling factor of 4×4.

For this study, we need to compare the performance of the same demosaicing algorithm using different CFA patterns. To make the CDMNet algorithm agnostic of the pattern, we made several modifications of the original version of CDMNet, shown in Figure 3 and explained in more details below:

1. We do not rely on an initial estimate obtained by bilinear interpolation. Any interpolation technique cannot guarantee equal reconstruction quality when applied on different CFA patterns. Since our goal is to compare the influence of the patterns on the reconstruction quality, it is necessary that all other conditions are equal when training the neural networks. Instead of initial interpolation, the network operates on the original, sub-sampled color channels provided at the input. To compensate for the lower spatial resolution of the input and reconstruct a high-resolution output, the idea of periodic shuffling is applied as explained in the next paragraph.
2. We integrated the idea of periodic shuffling in the CDMNet. Periodic shuffling was proposed for the problem of image super-resolution [42], in order to substitute the deconvolution layer for up-sampling. The effect of this operation is that the network operates on a lower spatial resolution, and interpolates the missing values in the feature channels. The number of channels is proportional to the sub-sampling factor. To obtain the final high-resolution output, the elements of the tensors of low spatial resolution and high dimensionality are re-arranged into a high-resolution RGB image.
3. All patterns used in these experiments were assumed to have the same size. Patterns that are smaller (e.g., 2×2 Bayer pattern or 4×2 Lukac pattern) can be considered as replicated in the appropriate dimension to achieve the largest size of all compared patterns, 4×4. The down-sampling factor *r* in the periodic shuffling is determined by the size of the pattern, and in this case we have fixed it to 4 in both the horizontal and vertical directions.

These adaptations allow for providing the same conditions when training the neural network for different patterns. Any difference in the reconstruction quality will only be a consequence of the pattern that was used. To train the neural network, we relied on the Waterloo Exploration Dataset (WED) [43] following the same practice as the original CDMNet method. Initially, we randomly select 4644 images to create the training dataset, and the remaining 100 images from WED are used as a validation set. We have then fixed the selected training and validation sets and used the same ones during re-training for all of the patterns.

Approximately 360,000 patches were extracted from the training images. Instead of the originally proposed patch size of 50×50, we extracted patches of size 48×48 to keep the dimensions divisible by the downscaling factor. For each pattern, the neural network was randomly initialized and re-trained for 81 epochs using batches of 64 patches. As in the original version [25], the learning rate was decreased five times every 20 epochs, ranging from $10^{-3}$ to $10^{-5}$. Applying the modified CDMNet model to any camera with a three-color, a repetitive (periodic) CFA pattern would require only one offline re-training and applying a scaling factor proportional to the pattern size.

## 4. Experimental Analysis

The objective of the performed analysis is to show that, as the performance quality increases with the more sophisticated reconstruction techniques being introduced, it becomes less affected by the CFA design.

### Experiments and Materials

In our study, we tested the two modified generic state-of-the art algorithms (that belong to different classes of algorithms) on Bayer-like patterns (where the sampling ratio between the green, red and blue channel is 2:1:1). We limit our analysis only to Bayer-like patterns, with a purpose of achieving fair comparison and to obtain unbiased (towards more sophisticated and novel CFA patterns) and uninfluenced (by different designs of demosaicing algorithms) conclusions. According to the performed analysis presented in [29], some random patterns perform better than Bayer for the specific LMMSE demosaicing methods. Our assumption is that the worst-performing CFAs (like Quad Bayer) will present the largest differences with respect to output quality as a function of the demosaicing algorithm used. Our experiments show that when neural networks are applied for demosaicing, the choice of the CFA pattern does not significantly influence the quality of the results. Based on our finding and the results of generic algorithms presented in [28] (which are similar for Bayer CFA and for Pseudo Randomized CFA), we expect that this trend will be true for random patterns as well. For this reason, the paper focuses on showing how the performance for the worst-performing CFAs, as these are the ones with the largest differences in performance, changes as a function of chosen demosaicing algorithm. Therefore, we test the hypothesis that if the exact CFA pattern becomes irrelevant or less relevant, this largest difference would diminish with it.

**Figure 4:**
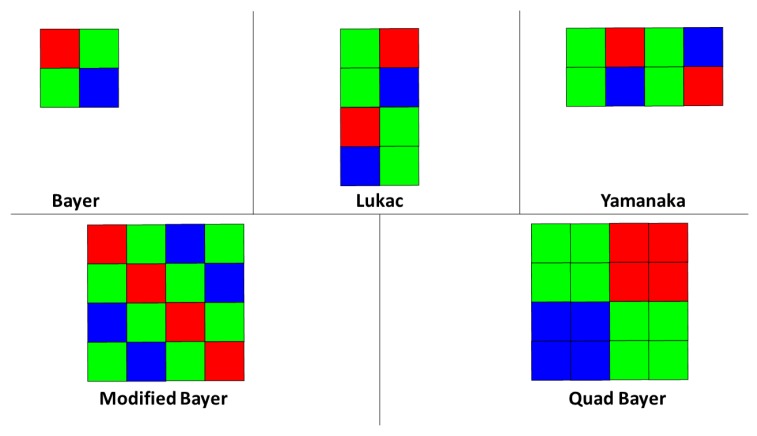
Bayer-like CFA patterns used in the experimental analysis: Bayer, Lukac, Yamanaka, Modified Bayer and Quad Bayer.

The patterns (see Figure 4) on which the modified state-of-the-art algorithms were tested are: Bayer pattern, Lukac pattern, Yamanaka pattern, Modified Bayer pattern and Quad Bayer pattern.

Following the common practice for quality performance evaluation, in the performed analysis for the two modified state-of-the-art generic algorithms, we used the two well-known image data-sets: KODAK [39] (abounding with highly diverse in content and with plenty of details images) consisted of 24 images in total (18 images with resolution of 768×512 pixels and six images with a resolution of 512×768 pixels) and IMAX [40] (abounding with highly diverse in content and colors and with plenty of details images) consisted of 18 images with resolution of 500×500 pixels. The second image data-set, IMAX, is more challenging for quality performance evaluation of the demosaicing algorithms. These image data-sets do not coincide with WED and are therefore new, previously unseen data for the modified CDMNet. For testing the quality performance of the modified CDMNet, each of the five trained neural network models (one model for every CFA design presented on Figure 4), was applied on the input mosaic images obtained with the appropriate CFA pattern.

In order to test our hypothesis that a generic (in terms of CFA pattern) *modern learning-based* demosaicing technique (such as the modified CDMNet) is more advantageous for reconstruction and less affected by the quality of the CFA pattern than a generic *classical* demosaicing technique that is not learning based (such as the modified ACUDe), we perform three experiments. The first two experiments are part of the quantitative analysis for the quality performance of the two algorithms and these include: objective evaluation (using the average CPSNR and the average PSNR for each color channel, with the standard deviation of the mean, calculated over the reconstructed images from each image data-set) and perceptual evaluation (using the SSIM metric, with the standard deviation of the mean calculated over the reconstructed images from each image data-set). When each quality metric was calculated, 11 pixels were excluded from the two vertical and horizontal image borders. In the third experiment, which is part of the qualitative analysis, we analyze and compare images that were reconstructed with the both algorithms, for the five different Bayer-like CFA patterns.

## 5. Results

In what follows, we present the results and the conclusions from the experiments of the performed analysis about the quality performance of the two representative state-of-the-art algorithms (the modified ACUDe as representative among the *classical* demosaicing algorithms and the modified CDMNet as representative among the *modern learning-based* demosaicing algorithms).

### 5.1. Results from the Quantitative Analysis

**Figure 5:**
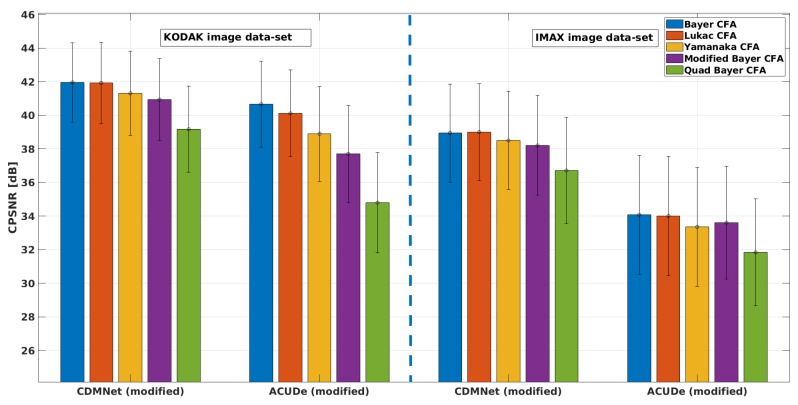
Average color peak signal-to-noise ratio (CPSNR) results with standard deviation of the mean: for the two modified generic algorithms (“CDMNet modified” and “ACUDe modified”), for the two data-sets (“KODAK” and “IMAX”), for five different Bayer-like patterns (Bayer, Lukac, Yamanaka, Modified Bayer and Quad Bayer).

**Figure 6:**
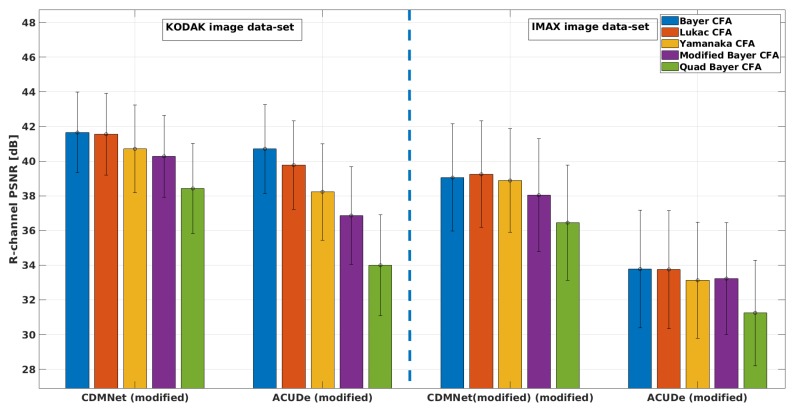
Average PSNR (red channel) results with standard deviation of the mean: for the two modified generic algorithms (“CDMNet modified” and “ACUDe modified”), for the two data-sets (“KODAK” and “IMAX”), for five different Bayer-like patterns (Bayer, Lukac, Yamanaka, Modified Bayer and Quad Bayer).

**Figure 7:**
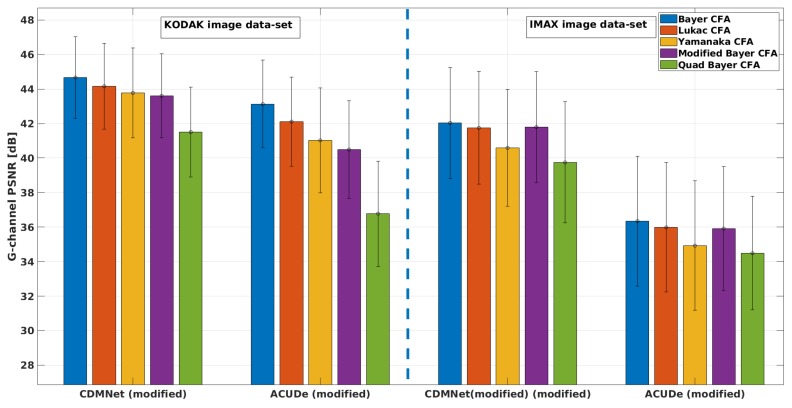
Average PSNR (green channel) results with standard deviation of the mean: for the two modified generic algorithms (“CDMNet modified” and “ACUDe modified”), for the two data-sets (“KODAK” and “IMAX”), for five different Bayer-like patterns (Bayer, Lukac, Yamanaka, Modified Bayer and Quad Bayer).

**Figure 8:**
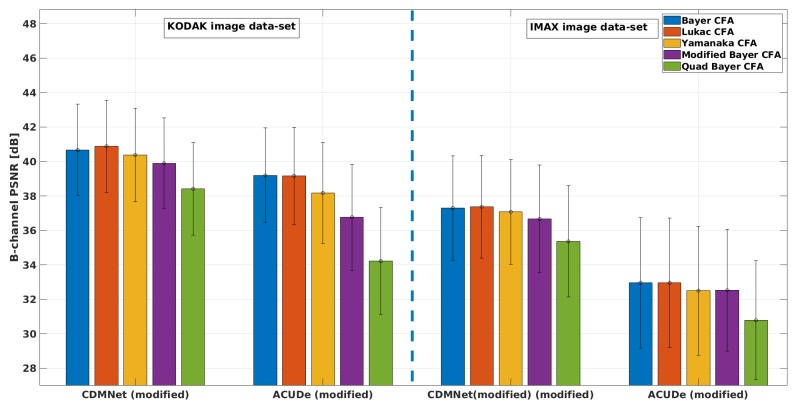
Average PSNR (blue channel) results with standard deviation of the mean: for the two modified generic algorithms (“CDMNet modified” and “ACUDe modified”), for the two data-sets (“KODAK” and “IMAX”), for five different Bayer-like patterns (Bayer, Lukac, Yamanaka, Modified Bayer and Quad Bayer).

**Figure 9:**
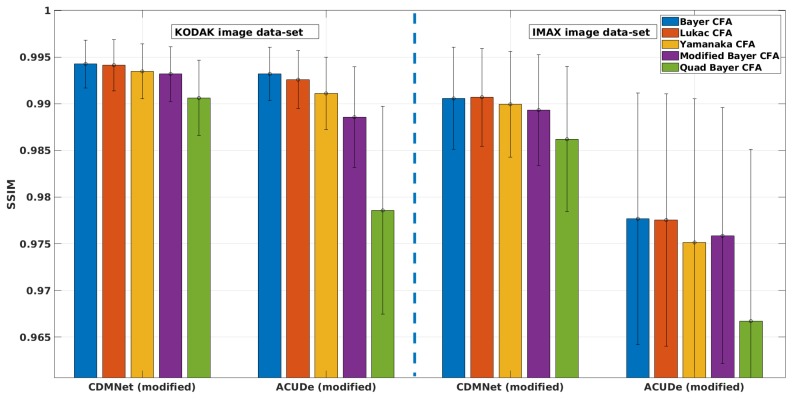
Average SSIM results with standard deviation of the mean: for the two modified generic algorithms (“CDMNet modified” and “ACUDe modified”), for the two data-sets (“KODAK” and “IMAX”), for five different Bayer-like patterns (Bayer, Lukac, Yamanaka, Modified Bayer and Quad Bayer).

In Figure 5, we present the averaged CPSNR results with the standard deviation of the mean (over the images from each image data-set) obtained for each analysed Bayer-like CFA pattern, with the representative techniques (the modified ACUDe and the modified CDMNet). In Figure 6, Figure 7 and Figure 8, we present the average PSNR results with the standard deviation of the mean (over the images from each image data-set) for each channel separately. In the same way, in Figure 9, we present the SSIM results.

From the CPSNR graph (see Figure 5), it can be noticed that the difference in quality, achieved with the modified CDMNet (especially on the KODAK image data-set) across the different Bayer-like CFA patterns, is smaller than the difference in quality of the modified ACUDe. The highest difference in the quality, which may be observed from the both PSNR and SSIM values and as it is expected, is between the Bayer CFA pattern and the Quad Bayer CFA pattern. The color pixels in Bayer CFA are more frequently distributed around the pixel of interest (the pixel whose value is to be estimated) and therefore the reconstruction quality with both algorithms is better for the Bayer CFA pattern. This difference (for the KODAK image data-set) of ≈3 dB when the modified CDMNet is applied, is significantly lower, when being compared to the ≈6 dB difference when the modified ACUDe is applied. Although not highly pronounced, a similar trend is recognized when the results of the IMAX image data-set are observed. The same conclusion can be derived from the observations on the PSNR results for each color channel separately (see Figure 6, Figure 7 and Figure 8).

To examine if this difference (between the reconstruction quality across the different CFA patterns) becomes more apparent when the modified ACUDe is applied, we proceed with analyzing the SSIM results (see Figure 9). As expected, the derived conclusion of the CPSNR and PSNR results is more notably supported with the SSIM results. This analysis brings us towards a more general conclusion on the improvement of the reconstruction quality, with the emergence of new and more advanced demosaicing techniques. The conclusion is that the reconstruction quality becomes less affected by the CFA pattern, as the demosaicing techniques become more sophisticated and more reliant on powerful deep learning-based approaches.

Although the absolute overall quality performance of both representative demosaicing algorithms (the modified ACUDe and the modified CDMNet) is not the main focus of the performed analysis, in what follows, with a purpose to make the analysis thorough, we will discuss the major differences between the two algorithms for each image data-set. The KODAK image data-set, compared to the IMAX image data-set, consists of natural images that are more color consistent. On the contrary, the IMAX image data-set abounds with images that consider more high spatial color frequencies. Therefore, the better quality performance of the both demosaicing algorithms, on the KODAK image data-set, is expected and justified. On the other hand, the color constancy in natural images is one of the assumptions that the design of many *classical* demosaicing techniques (also including the state-of-the-art algorithms: the generic ACUDe and ARI) is based on. Therefore, our assumption is that the significantly better quality performance of the modified ACUDe on the KODAK image data-set, rather than on the IMAX image data-set, is due to the fact that ACUDe, in the way it was originally designed, is inherently biased towards image content with higher color constancy. If the PSNR results for each color channel (see Figure 6, Figure 7 and Figure 8) are analyzed, it can be noticed that both algorithms are better in reconstructing the green color channel, rather than the blue and the red color channels. It can also be noticed that the difference in quality reconstruction between the content of the two image data-sets is smaller in the case when the modified CDMNet is applied. Moreover, the standard deviation of the mean (calculated over the reconstructed images from each image data-set and each analyzed CFA pattern) is smaller (especially when SSIM results are observed) in the case when the modified CDMNet is applied.

These results show that the modified CDMNet, as a representative among the *modern learning-based* demosaicing techniques, is more adaptive to different types of scenes and at the same time succeeds with achieving high quality reconstruction for different CFA patterns.

### 5.2. Results from the Qualitative Analysis

**Figure 10:**
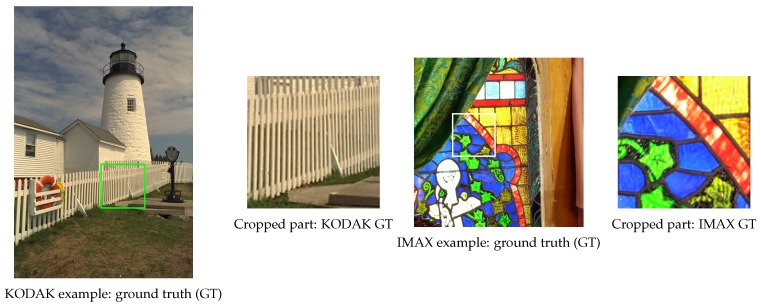
Examples of ground truth images from KODAK and IMAX image data-sets and their cropped parts.

**Figure 11:**
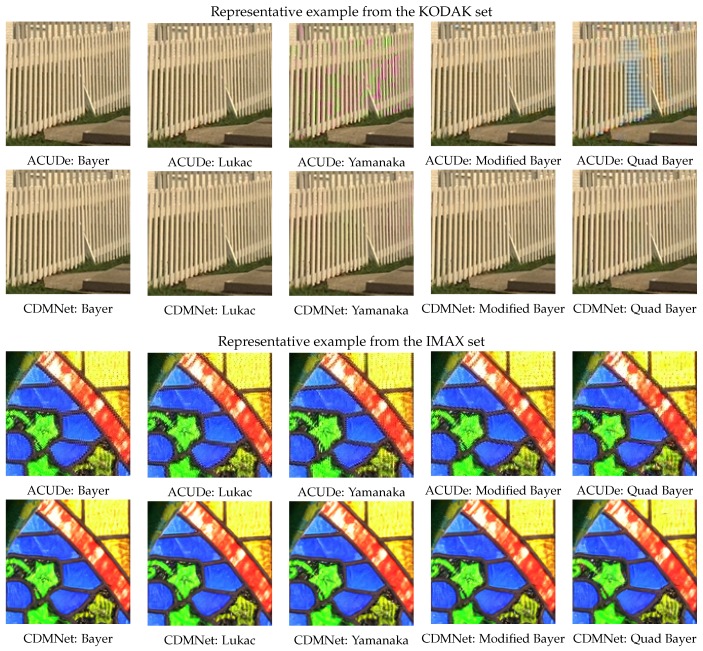
Results obtained for the representative examples from KODAK and IMAX data-sets. Two algorithms were analysed: (1) the modified ACUDe based on the algorithm presented in [28], as a state-of-the-art generic algorithm among the *classical* demosaicing techniques and (2) the modified CDMNet based on the algorithm presented in [25] as a state-of-the-art generic algorithm among the *learning-based* demosaicing techniques. The algorithms were tested on five Bayer-like CFA patterns. There are no big visual differences between the reconstructed images from different CFA inputs when the modified CDMNet (the representative of the *modern learning-based* demosaicing techniques) is applied.

Here, we visually present the results (cropped parts of the reconstructed images) from the selected representative examples of each image data-set (KODAK and IMAX). The ground-truth (GT) images, with the corresponding cropped parts (marked with rectangles), are presented in Figure 10. In Figure 11, we show the results obtained with the both algorithms (the modified ACUDe and the modified CDMNet) for the five Bayer-like CFA patterns presented in Figure 4. The differences between the reconstructed images from the different CFA inputs are visually more pronounced in the case when the modified ACUDe is applied. Note the color aliasing artifacts in the result for the KODAK example and the Yamanaka CFA pattern and the color aliasing artifacts accompanied with zippering artifacts for the KODAK example and the Quad Bayer CFA pattern presented in Figure 11. Some color aliasing artifacts may also be noticed (although negligible) in the reconstructed images with the modified CDMNet (see the result obtained with the modified CDMNet, for the KODAK example and the Yamanaka CFA pattern, presented in Figure 11). If we compare the results for the Quad Bayer CFA pattern and the Bayer CFA pattern, obtained with the modified CDMNet, we notice that there are no demosaicing artifacts present. The only difference between the two results may be perceived as insignificant blurriness in the case of the Quad Bayer CFA pattern. Furthermore, if the results obtained with the modified CDMNet for the IMAX example are observed, almost no differences between the reconstructed images will be noticed.

The consistently higher reconstruction quality across different patterns and data-sets achieved by CDMNet, over ACUDe, can be attributed to the strong representation power of convolutional neural networks. The advantage of CNN models compared to classical methods is the capability of modeling a distribution of natural images, and learning spatial and spectral correlations in the data. The huge number of trainable parameters in CDMNet provides sufficient model complexity for solving the demosaicing problem with similar quality for different input mosaic configurations.

The presented results from the performed experimental analysis, additionally with the results from the experimental evaluation presented in [30], veritably support our initial hypothesis that, when *modern learning-based* techniques are applied for demosaicing, the overall reconstruction quality is less influenced by the choice of the CFA pattern.

## 6. Conclusions

Within this study, we analyse the quality performance of two state-of-the-art generic demosaicing algorithms for different Bayer-like CFA patterns. The first one (modified ACUDe) belongs to the group of *classical* demosaicing algorithms, while the second one (modified CDMNet) belongs to the group of *modern learning-based*, i.e., neural-network-based demosaicing algorithms. The aim of the performed analysis is to test the hypothesis that the *modern learning-based* demosaicing techniques (the NN-based approaches) overcome the high difference in quality performance for different CFA patterns and at the same time succeed at achieving quality performance that is higher than the quality performance of the state-of-the-art generic algorithm (here modified ACUDe) that belongs to the group of *classical* demosaicing techniques. The presented results of the analysis for the quality performance indeed show that the hypothesis is true. From this study, we derive a conclusion about the constantly increasing reconstruction power of the *modern learning based* demosaicing algorithms towards adaptiveness to any CFA design without loss in the reconstruction quality (which used to be dependent on the quality of the CFA design). This conclusion leads to a finding regarding the future opportunities for camera manufacturing and image reconstruction, specifically in combining lower hardware requirements with powerful reconstruction techniques. In other words, this means that, with the *modern learning-based* demosaicing methods, camera manufacturers have more freedom in the choice of the CFA pattern layout, without a noticeable loss in the image quality. In that direction, the patterns can be adapted to improve other image properties and facilitate various imaging tasks, such as the Quad Bayer that was designed to improve noise reduction in low-light imaging. Furthermore, this conclusion points towards the advantage of using the easily adaptive and retrainable neural-network based demosaicing techniques in various applications of multispectral imaging.
